# Effects of exercise-targeted hippocampal PDE-4 methylation on synaptic plasticity and spatial learning/memory impairments in D-galactose-induced aging rats

**DOI:** 10.1007/s00221-023-06749-9

**Published:** 2023-12-05

**Authors:** Yu Jin, Xue Li, Changling Wei, Qiongjia Yuan

**Affiliations:** School of Sport Medicine and Health, Chengdu Sport University, Chengdu, 610041 China

**Keywords:** Exercise, Phosphodiesterases-4, Methylation, Spatial learning/memory, Brain aging

## Abstract

Physical exercise reduces the effects of aging and cognitive decline by improving synaptic plasticity and spatial learning. However, the underlying neurobiological mechanisms are unclear. A total of 45 Male SPF Sprague–Dawley rats were acclimatized and then allocated into three groups, 15 in each group: the saline control (DC) group, D-gal-induced aging (DA) group, and D-gal-induced aging + exercise (DE) group. Six weeks of intraperitoneal injections of D-gal at a concentration of 100 mg/kg body weight/d was injected to establish model of aging in the DA and DE groups. Morris water maze test was implemented to evaluate the hippocampus related cognition. SOD activity and MDA was tested to assess the aging in all groups. H&E and Nissl staining was used to observe the histopathological changes of hippocampal neurons in aging rats. Quantitative real-time polymerase chain reaction, western blotting and immunofluorescence staining techniques were used to investigate the expression of synaptic genes and proteins in the hippocampus. Massarray methylation system was employed to measure the PDE-4 gene methylation level in rat hippocampal tissues. Our results demonstrated that exercise intervention improves cognitive function in D-gal-induced aging rats. The methylation of CpG sites in PDE-4 in the hippocampus was significantly increased. The physical exercise significantly increased PDE-4 gene methylation and effectively decreased PDE-4 gene and protein expression. These beneficial behavioral and morphological effects were attributed to PDE-4 methylation, which was activated cAMP/PKA/CREB pathway and improved synaptic plasticity. Exercise induced PDE-4 methylation is key mechanism underpinning the amelioration of learning/memory impairment, suggesting the potential efficacy of physical exercise training in delaying brain aging.

**Supplementary Information:**

The online version contains supplementary material available at 10.1007/s00221-023-06749-9.

## Introduction

Aging is the most significant risk factor for human chronic diseases, including cardiovascular diseases, metabolic diseases, musculoskeletal diseases, neurodegenerative diseases, and cancer (Song et al. 2020). The United Nations has reported that between 2019 and 2050, the number of persons aged 65 or over worldwide is projected to more than double to over 1.5 billion, accounting for 16% of the world population (Partridge et al. 2018; Cho and Stout-Delgado 2020; Nation 2020). As the brain is one of the organs that is most affected by aging in humans, brain aging can lead to cognitive impairment and increase the risk of neurodegenerative diseases. The hippocampus which is the part of brain, mainly responsible for the cognitive function, and the regulation of emotional behavior. The hippocampal structure and function is susceptible to the aging process, as the aging-related hippocampal atrophy is caused by neuronal loss and impaired neurogenesis. The cognitive impairment caused by aging is a significant challenge for policymakers, healthcare institutes, and families. However, effective treatments for aging-related neurodegenerative diseases have not been developed thus far. Therefore, maintenance of brain health throughout life has become a hot social issue that has attracted much attention, and effective ways for addressing this issue are urgently needed. Several animal models have been developed to investigate the processes of aging-related cognitive impairment. In this study, D-galactose-induced aging model rats were utilized to investigate whether exercise-induced PDE-4 methylation improved hippocampus synaptic plasticity and spatial learning.

Exercise is beneficial for the structure and function of the brain, especially in older adults, and can help restore cognitive functions, including hippocampus-dependent spatial learning/memory ability, and maintain brain function (Tyndall et al. 2018; Spartano et al. 2019). As a critical brain area for learning and memory consolidation, the hippocampus exhibits increased oxidatively damaged molecules and inflammation, intracellular signal transduction, changes in gene expression, decreased neurogenesis, and synaptic plasticity, which are related to age-induced changes in cognitive function (Bettio et al. 2017). Numerous signaling pathways are stimulated by exercise, including the cAMP-PKA signaling pathway, which prevents osteoporosis (Peng et al. 2022), and the CREB signaling pathway, which promotes antidepressant effects (Kim and Leem 2014). Similarly, exercise inhibits lipopolysaccharide-induced inflammation in cultured macrophages and myocardial hypoxia/reoxygenation-induced apoptosis via the S1P/cAMP/Akt signaling pathway (Otaka et al. 2018). Exercise or enriched environment (EE) stimulates cAMP/PKA signaling and induces hippocampus synaptic plasticity by activating β-adrenoceptor signaling and mitigating synaptotoxicity of human Aβ oligomer (Li et al. 2013). The expression of cAMP-dependent PDE4 including some other non-dependent PDE genes (PDE1, PDE2, and PDE3) was increased by exercise (Han et al. 2018). cAMP-specific PDE4 is abundantly expressed in the brain, cardiovascular tissues, smooth muscles, keratinocytes, and immunocytes (T cells, monocytes, macrophages, neutrophils, dendritic cells, eosinophils) (Chiricozzi et al. 2016). PDE4 inhibition increases intracellular cAMP and modulates inflammatory responses and immunological homeostasis (Maurice et al. 2014).

Phosphodiesterase-4 (PDE-4) plays an indispensable role in memory consolidation and retention. It has been used as a vital target for the development of new drugs for the treatment of dementia and Alzheimer’s disease (AD) (Kumar et al. 2015; Prickaerts et al. 2017; Ju and Tam 2022). Several academic studies have established that inhibition of PDE-4 expression in the hippocampus can directly lead to activation of the cAMP/PKA/CREB signal and boost learning and memory (Epp et al. 2007; Cheng et al. 2010; Titus et al. 2013). cAMP is involved in synaptic transmission, neuronal excitability, neuroplasticity, and neuroprotection, and it can exert its effects through the cAMP/PKA/CREB signaling pathway in the hippocampus (Heckman et al. 2018; Yuan et al. 2021). cAMP/PKA/CREB pathway initiate the production of growth factors such as brain-derived neurotrophic factor (BDNF) and BDNF with its receptor TrkB controls the trafficking of PSD-95 (Yoshii and Constantine-Paton 2014), which has been identified as a marker for synaptic strength. Inhibition of PDE-4 expression can be achieved through DNA methylation via epigenetics and is a novel mechanism by which exercise may delay the symptoms of brain aging and neurodegenerative diseases (Kader et al. 2018).

Aging is the main risk factor for cognitive decline; hence it is important to identify the epigenetic changes linked to age-related cognitive decline. The PDE-4 inhibitor roflumilast was found to improve language learning in young adults and elderly healthy volunteers in humans (Blokland et al. 2019b). Therefore, PDE4 inhibition appears to have therapeutic promise as a remedy to improve memory performance. The discovery that PDE-4 inhibition can reverse memory deficits brought on by intra-hippocampal injections of amyloid-β in rats supports the therapeutic promise in AD (Cheng et al. 2010). Until now, four isoforms (PDE4A, PDE4B, PDE4C, and PDE4D) of PDE-4 have been discovered and all isoforms can hydrolyze cAMP and are expressed in the brain and have neuroprotective effects after inhibition (Blokland et al. 2019a). Thus, the aim of current research was to evaluate the effects of a 6-week exercise training on PDE-4 gene methylation, synaptic plasticity, and spatial learning/memory. We used the MassArray system to examine PDE-4 DNA methylation in a rat model of aging induced by D-galactose (D-gal) administration for 6 weeks during exercise. Molecular experiments and protein expression analysis were performed to identify the molecular mechanism underlying the effect of exercise training. The results revealed that inhibition of PDE-4 expression by DNA methylation, promotion of cAMP/PKA/CREB pathway-related protein expression and improvements in synaptic plasticity are the critical mechanisms by which physical exercise prevents and alleviates cognitive impairments caused by aging.

## Materials and methods

### Experimental design and exercise protocol

A total of 45 Male SPF Sprague–Dawley rats (3-month-old) were bought from the Experimental Animal Centre of Chengdu Dashuo Biological Technology Co., Ltd. and were housed under SPF conditions at the center. The rats were given free access to standard feed and water and housed on a 12 h of light and dark cycle. After 1 week of acclimatization, the rats were randomly divided into three groups, 15 in each group: the saline control (DC) group, D-gal-induced aging (DA) group, and D-gal-induced aging + exercise (DE) group. Aging was induced in rats in the DA and DE group by intraperitoneal injection of D-gal at a dose of 100 mg/kg body weight/d for six weeks. Rats in the DC group were injected with the same volume of saline. Furthermore, swimming was selected as the aerobic exercise mode for the DE group in a transparent glass jar (160 cm*60 cm*110 cm) with a water depth of 80 cm and a temperature of 32 ± 2 °C (Li et al. 2019). The exercise protocol was performed for 60 min/day six days/week for six weeks. The Animal Ethics Committee (Batch No: 2021–13) of Chengdu Sport University approved all animal experiments.

### Morris water maze (MWM) test

The MWM test was performed as previously reported (Chen et al. 2020). The MWM apparatus consisted of a water-filled circular pool filled with water. In one quadrant, a platform was placed 1 cm below the surface of the water. The MWM test was performed over six days, included the navigation phase (1st–5th days) and spatial exploration phase (6th day). For first five consecutive days, four trials per day were conducted and during each trail rats were placed in the water in different quadrants and allowed to find platform. If a rat failed to find the correct platform within 120 s, it was slowly directed to the platform and allowed to stay on it for 10 s. The escape latency of each rat within 120 s was recorded. The average escape latency to platform of the rats was been calculated. The platform was removed on the sixth day, and the swimming paths of the rats and the number of first platform crossings within 60 s were recorded. The animal behavior analysis system and the MWM video analysis system were provided by Anhui Huaibei Zhenghua Biologic Apparatus Facilities.

### Enzyme-linked immunosorbent assay (ELISA)

The SOD activity in brain homogenates was measured by SOD-ELISA kit (mlbio, Shanghai, China). The expression of MDA was measured by MDA-ELISA kit (mlbio, Shanghai, China). The level of cAMP in the hippocampus was quantified by using an cAMP-ELISA kit (mlbio, Shanghai, China) according to the manufacturer’s protocol. After the color development, the absorbance was measured at 450 nm with a fluorescence reader (Thermo, USA).

### Hematoxylin and eosin (H&E) and nissl staining

H&E staining was performed according to conventional methods (Chen et al. 2020). Rat brain tissues were perfused with normal saline followed by 4% paraformaldehyde (PFA) solution for 24 h. Then the tissues were paraffin-embedded, sectioned at 5-μm thickness (RM2016, Leica), and stained with H&E or toluidine blue. Coronal brain sections were used for H&E and Nissl staining, and the morphological structure of hippocampal neurons was observed under a light microscope. Individual cell number in the CA3 region was quantified using ImageJ software.

### RT-qPCR

Total RNA was extracted from hippocampal tissue using TRIzol (Invitrogen) according to the manufacturer's instructions. The concentration and purity of total RNA were determined, and RNA integrity was examined by agarose gel electrophoresis. cDNA was synthesized by reverse transcription using the BIO-RAD iScriptTM cDNA Synthesis Kit. RT-qPCR was performed using the SYBR Green method. The primer sequences for PDE-4 and the internal reference gene β-actin (TaKaRa) are shown in Table 1. The relative gene expression was calculated using the 2^−∆∆CT^ method (CT = threshold cycle).

**Table 1:** The primer sequences for PDE-4 and the internal reference gene β-actin

Gene	Application	Primer (5ʹ–3ʹ)
*PDE-4*	RT-qPCR	F: AGGAGAAATCAGCGTTGGAGA
		R: TTGTAGATGGTGAGGGTAGAGGA
*β-Actin*	RT-qPCR	F: CGTAAAGACCTCTATGCCAACA
		R: TAGGAGCCAGGGCAGTAATC
*PDE-4*	Methylation	F: AGGAAGAGAGTTTATGAAATATTTATGAGGGTTTTTGA
		R: CAGTAATACGACTCACTATAGGGAGAAGGCTAAAATATAAAAACTTCAACAATTCAACTC

### Western blot analysis

After carefully removing the brain, the hippocampus was quickly dissected on ice. Then tissue samples were lysed using RIPA lysis solution (containing PMSF), and the protein concentration was determined using BCA Protein Assay Kit (Beyotime, Shanghai, China). Equal amounts of total protein were mixed with loading buffer and boiled for 10 min. Then the proteins were separated on 10% SDS-PAGE and transferred onto polyvinylidene difluoride membranes. The membranes were then blocked for 2–3 h at room temperature (RT) with TBST buffer containing 5% skimmed milk. The blots were incubated overnight at 4 °C with the following antibodies: PDE-4 (ab14628, Abcam, 1:1000), PKA (ab75991, Abcam, 1:1000), CREB (ab32515, Abcam, 1:1000), PSD-95 (ab18258, Abcam, 1:1000), GAPDH (AF7021, Affinity, 1:3000) and β-actin (AF7018, Affinity, 1:3000). The membranes were further incubated for 2 h at RT with horseradish peroxidase-conjugated antibody, IgG (H + L) (S0001, Affinity, 1:5000) diluted in TBST containing 5% skimmed milk. After washing, the protein bands were finally visualized using an imaging system. The integrate gray value of each band was measured using ImageJ (National Institutes of Health, Bethesda, USA) to analyze the relative expression of each protein.

### Immunofluorescence staining

Rat brain tissue sections (RM2016, Leica) were embedded in paraffin. The paraffin sections were dewaxed and subjected to antigen retrieval and blocked with 5% BSA at RT for one hour. Then the sections were incubated with primary antibodies synaptophysin (Syp; 36,406, CST, 1:200) overnight at 4 °C. Subsequently, the sections were washed with PBS and incubated with secondary antibody (BA1090, Boster, 1:400) at RT for two hours. Then again washed with PBS, sealed with a drop of mounting medium (containing DAPI) and placed in an oven at 37 °C for 20 min. The sections were photographed under a fluorescence microscope (Imager Z2, Zeiss, Germany). The average fluorescence intensity was analyzed using ImageJ software.

### Massarray methylation assay

The PDE-4 gene methylation level in rat hippocampal tissues was measured by time-of-flight mass spectrometry. The CpG site was concentrated at residues 60–502 of PDE-4, and this fragment was selected as the target sequence. Primers were designed using EpiDesigner software. The primers for methylation analysis of the PDE-4 gene are shown in Table 1. Total DNA was extracted from hippocampal tissue, 200 µL of tissue lysis solution and 40 µL of proteinase K were added, and the samples were placed in a water bath at 55 °C to lyse them fully. Then, 200 µL of binding buffer was added, the samples were placed in a 70 °C water bath to allow full binding, 100 µL of isopropanol was added, the samples were shaken well, and the supernatant was collected. The DNA concentration and DNA purity were assessed by agar gel electrophoresis and measurement of optical density values, respectively. Amplified PCR reagents were detected by methylation using the Sequenom MassArray system, and then PCR was performed. The SAP reaction was then performed, and the T-cut reaction/RNase A precipitation was followed by desalting and resin purification. DNA methylation was quantified at the target site of the PDE-4 gene using MassARRAY^®^ EpiTYPER^™^ software that came with the mass spectrometry methylation detection platform.

### Statistical analysis

The one-way analysis of variance (ANOVA) and Tukey post hoc test were used to compare differences between two or more groups, respectively. Two-way ANOVA and Tukey post-hoc comparison were employed for time-dependent analysis. All of the results are shown as the mean ± standard deviation (SD). *P* < 0.05 was considered significant. All statistical analyses were performed with GraphPad Prism 8.0 (La Jolla, CA, USA). *, **, and ***indicate *P* < 0.05, *P* < 0.01, and *P* < 0.001, respectively.

## Results

### Exercise attenuated spatial learning/memory dysfunction in a rat model of D-gal-induced aging

**Fig. 1:**
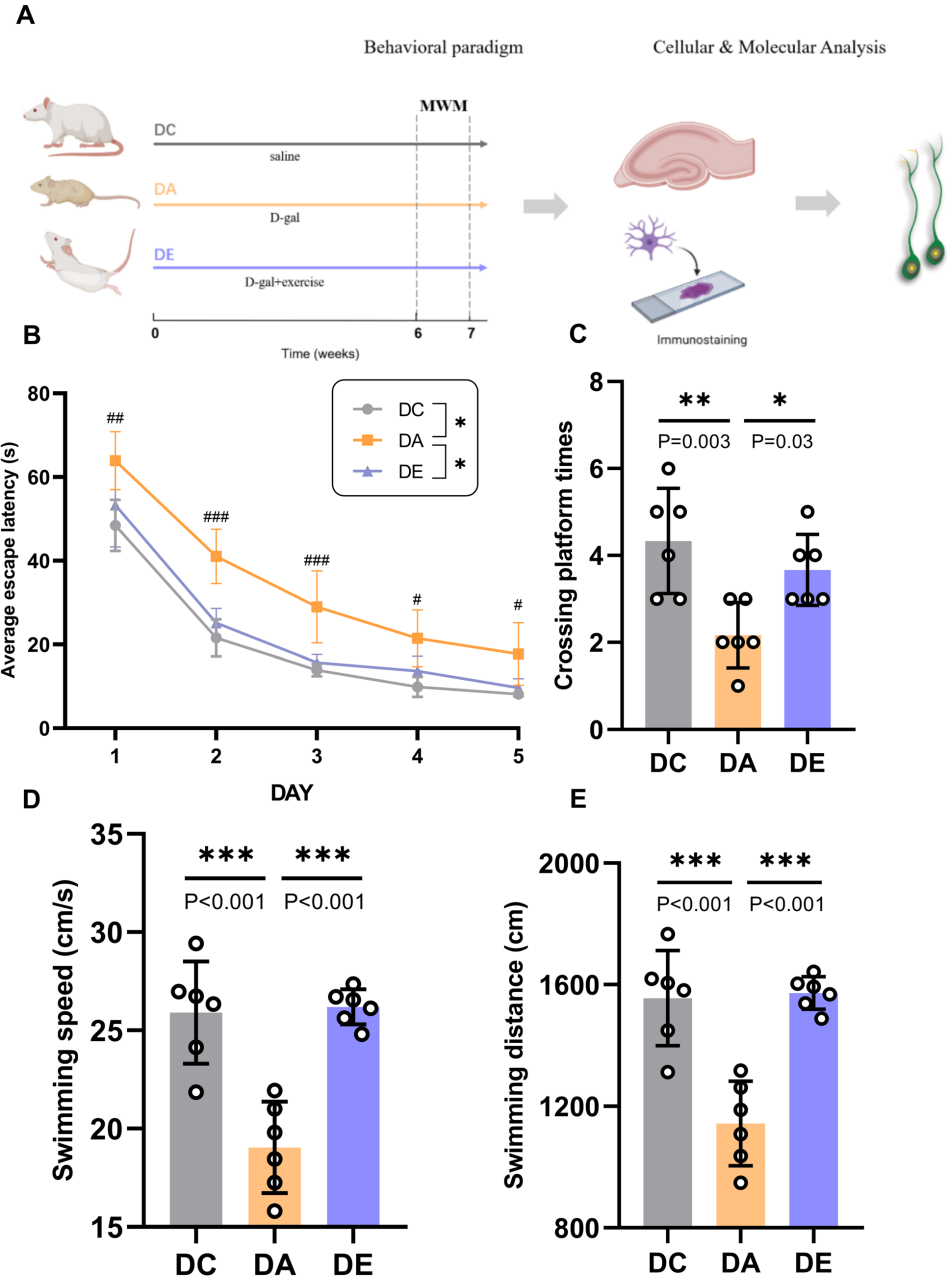
Exercise Attenuated Spatial Learning/Memory Dysfunction. **A** Schematic diagram for experimental protocols. DC group, injected with saline for six weeks; DA group, injected with D-gal for six weeks to establish a rat model of aging; DE, injected with D-gal for six weeks while undergoing aerobic exercise. **B** Escape latency to reach the platform on 1–5 days in the MWM test (n = 6). **C** The number of target platform crossings on the 6th day of the MWM test (n = 6). **D** The swimming speed of the rats on the 6th day (n = 6). **E** The swimming distance on the 6th day in the MWM test (n = 6). *, **, and *** indicate *P* < 0.05, *P* < 0.01, and *P* < 0.001, respectively. #to mark the comparison between DA and DE

Rats were injected with D-gal for six weeks and subjected to swimming exercise (Fig. 1A). After exercise, the MWM test was performed to assess hippocampus-dependent spatial learning and memory ability (Wang et al. 2020) to reveal the potential neuroprotective effects of exercise. The average escape latency of each group decreased during the training days. The escape latency of the DA group was longer than that of the DC and DE groups from days 1–5 (Fig. 1B, *P* < 0.05*, P* < 0.05). On the 6^th^ day, the number of original platform crossings was used as a measure of memory. As shown in Fig. 1C, the DA group had significantly fewer target platform crossings than the DC and DE groups (Fig. 1C , *P*= 0.003, *P* = 0.03), indicating that the DA group exhibited deficits in spatial learning and memory. Exercise also increased the swimming speed and distance in the MWM test (Fig. 1D, E, *P* < 0.001).

### Exercise mitigated damage to hippocampal neurons in aging rats

**Fig. 2:**
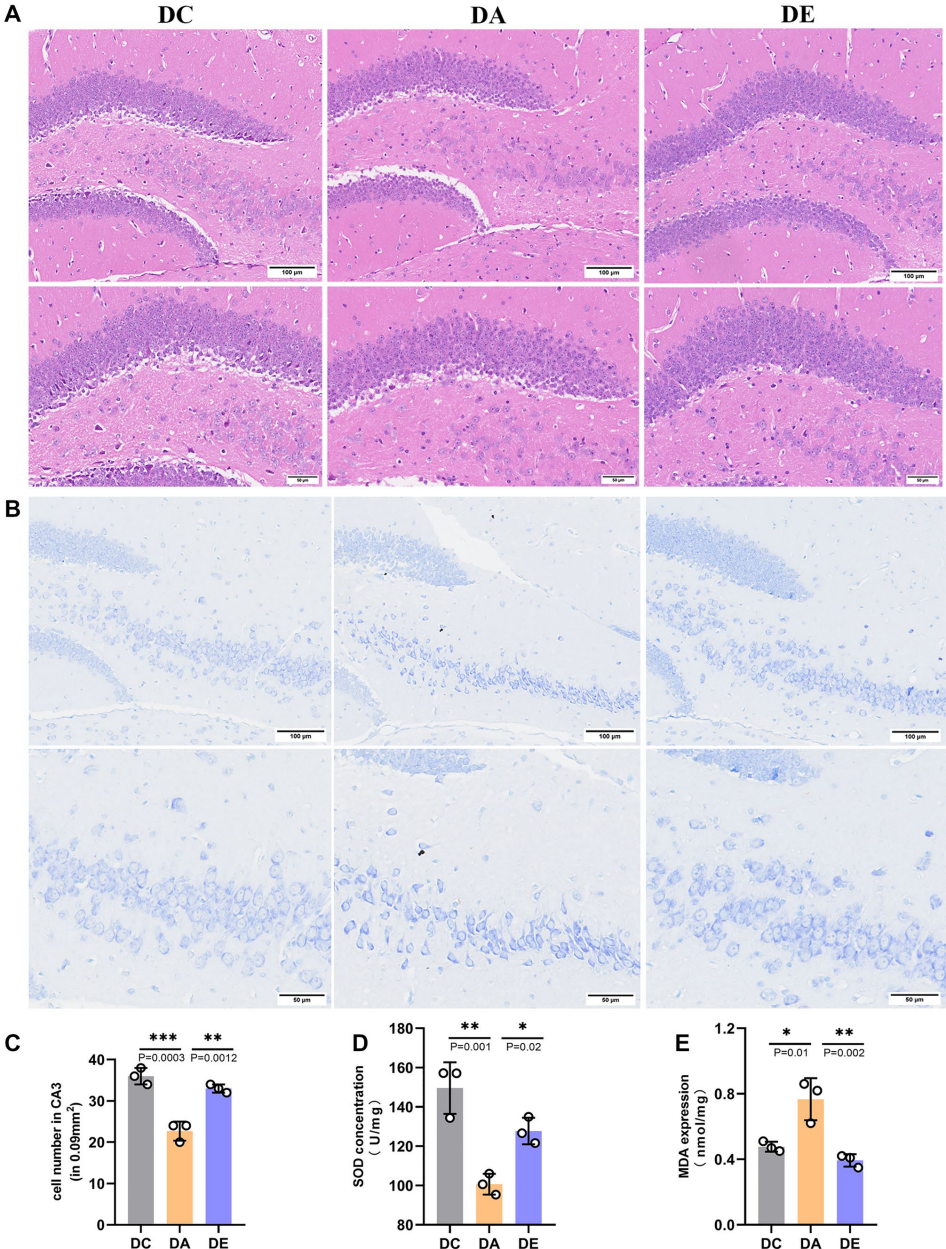
Exercise Mitigated Damage to Hippocampal Neurons in Aging Rats. **A**, **B** Representative photomicrographs demonstrating histopathological changes in hippocampal tissues (200 × , 400 ×) with H&E staining in DG (**A**) and Nissl staining in CA3 (**B**) [scale bar, 100 μm, 50 μm]. **C** The number of neurons was quantified in the hippocampus with Nissl staining in CA3 (n = 3 from three animals in each group, area = 0.09 mm^2^). **D** SOD concentrations were determined by ELISA (n = 3). **E** MDA expression were determined by ELISA (n = 3). *, **, and ***indicate *P* < 0.05, *P* < 0.01, and *P* < 0.001, respectively

We observed histopathological changes by H&E and Nissl staining to evaluate the damage to hippocampal neurons in aging rats. In the DA group, neurons in the hippocampal dentate gyrus (DG) were decreased in number than in the DC group. On the other hand, hippocampal neurons in the DE group were markedly increased in number, and the neuronal damage or loss caused by aging was reversed. Aerobic exercise significantly decreased hippocampal neuron damage, indicating that aerobic exercise can effectively reduce the hippocampal tissue damage (Fig. 2A). Moreover, compared with the rats from DC group, the neurons in hippocampal CA3 subfield of the rats from DA group were markedly decreased and damaged or lost in hippocampal tissue. The DE group subjected to exercise training inhibited a significant decrease the number of neurons, suggesting that exercise interventions can effectively attenuate the damage of hippocampal tissues in aging rats (Fig. 2B, C, *P* < 0.01). In addition, Superoxide dismutase (SOD), the primary endogenous enzymatic defense system of all aerobic cells, was considerably reduced in the DA group compared to the DE and DC groups (Fig. 2D). Malondialdehyde (MDA), an index of lipid per oxidation, indicates the overproduction of ROS (Dias-Santagata et al. 2007; Xu et al. 2020) was decreased the level of MDA in the aging brain by exercise (Fig. 2E). These results indicated that physical exercise can efficiently enhance the antioxidant enzyme in brain during aging.

### Exercise promoted PDE-4 methylation and inhibited PDE-4 expression in the hippocampi of aging rats

**Fig. 3:**
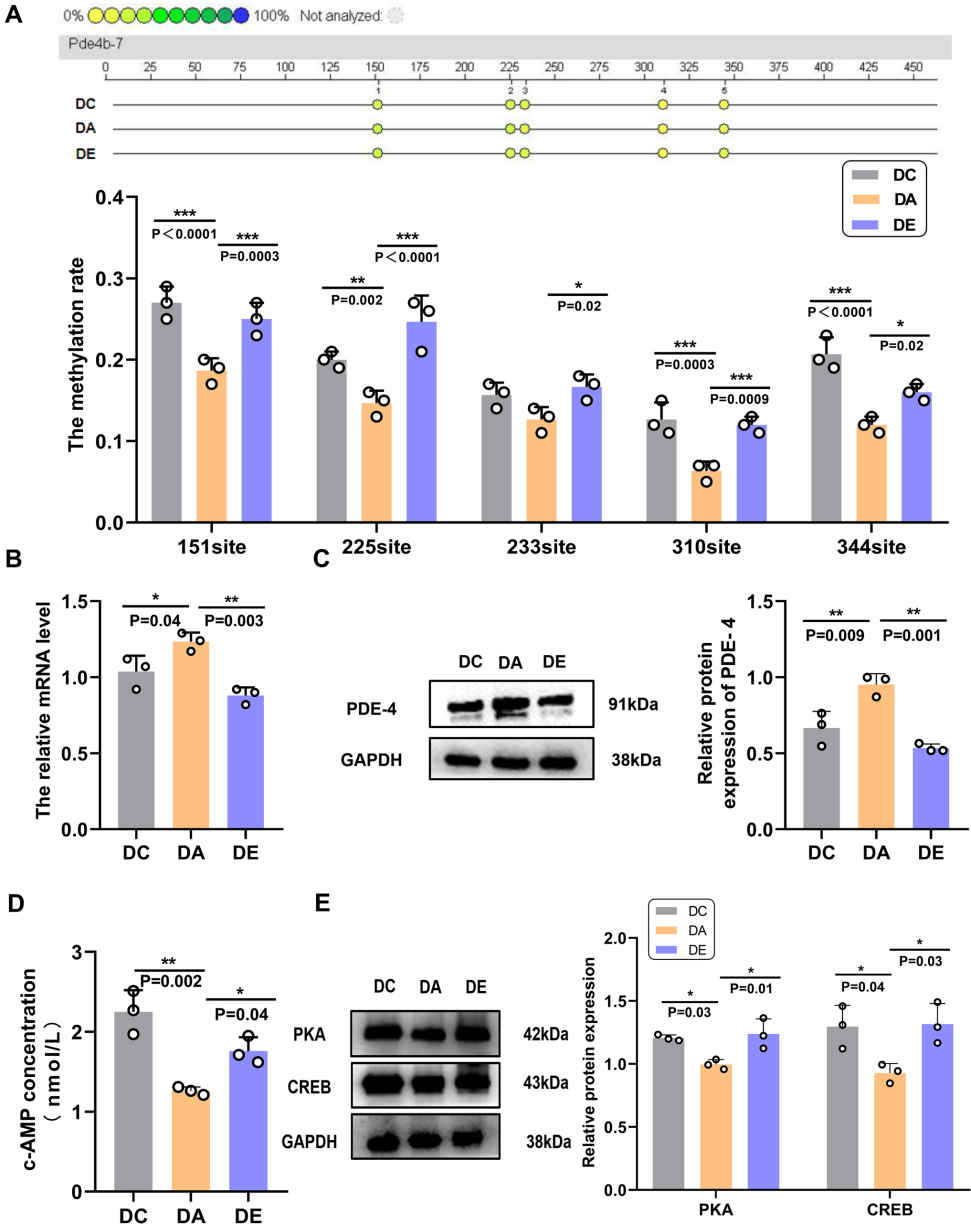
Exercise Promoted PDE-4 Methylation and Inhibited PDE-4 Expression. **A** The methylation sites of the PDE-4 gene in different groups (n = 3). **B** The mRNA expression of PDE-4 in the hippocampus (n = 3). **C** The relative protein expression of PDE-4 in the hippocampus and the corresponding protein band. **D** cAMP levels in the hippocampus, as determined by ELISA (n = 3). **E** The relative protein expression of PKA and CREB in the hippocampus (n = 3). Representative western blotting bands. *, **, and ***indicate *P* < 0.05, *P* < 0.01, and *P* < 0.001, respectively

MassArray system was employed to examine PDE-4 gene methylation. The results revealed that the methylation of CpG sites in PDE-4 in the hippocampus was significantly increased in the DE group than in the DA group. We observed substantial DNA hypermethylation at five PDE-4 CpG sites, including the 151, 225, 233, 310, and 344 sites (Fig. 3A, *P* = 0.0003, *P* < 0.0001, *P* = 0.02, *P* = 0.0009, *P* = 0.02).

Then, we performed RT-PCR and WB to examine the gene and protein expression of PDE-4. After six weeks of exercise training the mRNA and protein expression of PDE-4 was downregulated in the hippocampus (Fig. 3B, *P* = 0.003; Fig. 3C, *P* = 0.001), demonstrating that regular exercise inhibited PDE-4 expression. PDE-4 inhibitors have previously been shown to increase synaptic plasticity via the cAMP/PKA/CREB signal pathway and restore cognitive impairments (Kelly 2018; Schreiber et al. 2020). We used ELISA to measure cAMP expression in the hippocampus, and the results showed that the DA group had significantly lower cAMP level than that the DC group (Fig. 3D, *P* = 0.002). After intervention for six weeks, the level of cAMP in the hippocampus was increased (Fig. 3D, *P* = 0.04). Furthermore, PKA and CREB expression was significantly higher in rats subjected to exercise compared with rats in the DA group (Fig. 3E, *P* = 0.01, *P* = 0.03). Exercise training significantly increased PDE-4 methylation and effectively decreased PDE-4 gene and protein expression. Subsequently, the down-regulation of the PDE-4 expression promotes the cAMP/PKA/CREB pathway by enhancing their expression level.

### Exercise increased synaptic plasticity in the hippocampi of aging rats

**Fig. 4:**
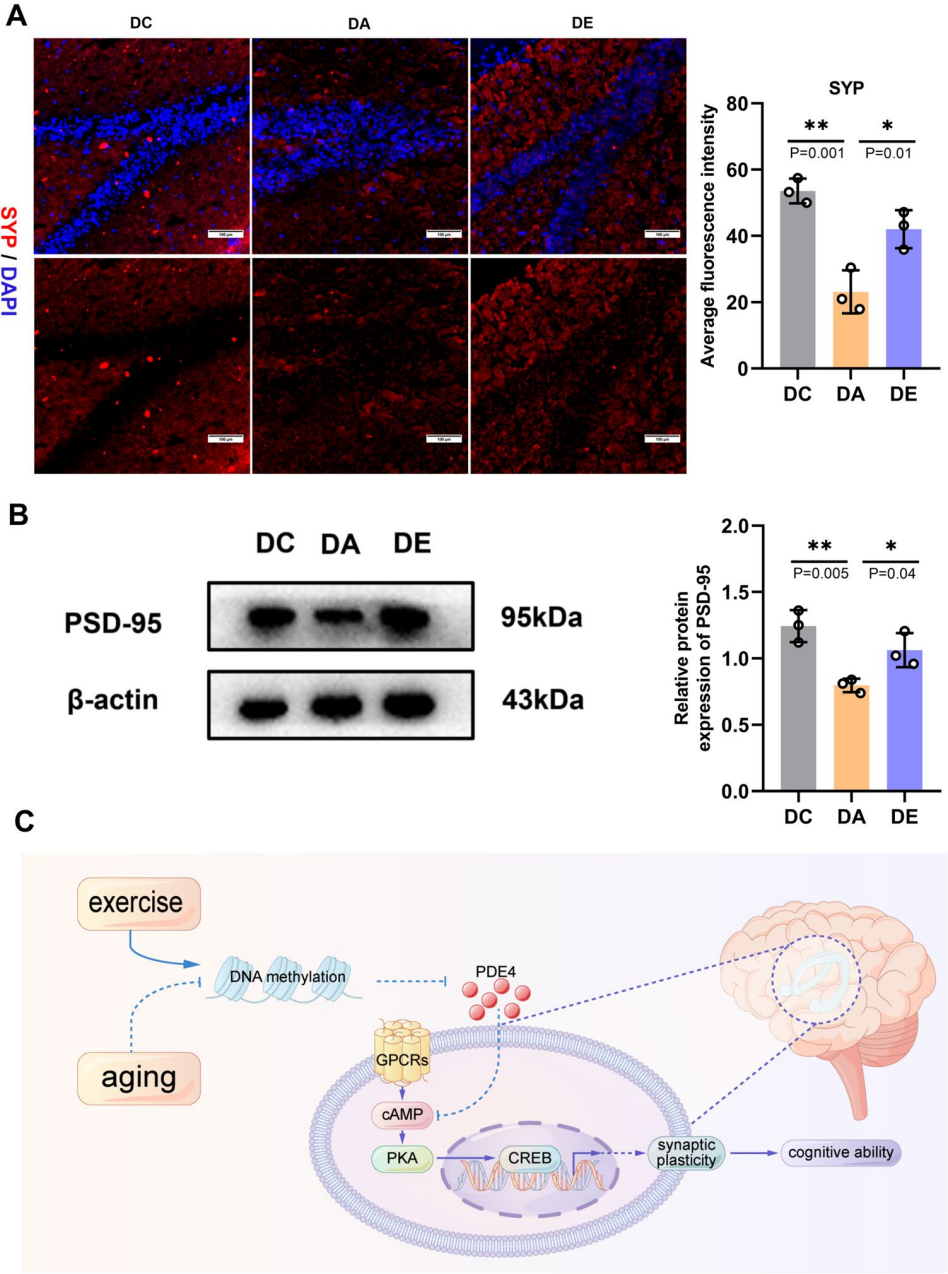
Exercise Increased Synaptic Plasticity in the Hippocampus. **A** Immunostaining of SYP (synaptophysin) protein expression (200 × magnification, scale bar, 100 μm) in the hippocampus, the average fluorescence intensity was measured of DC, DA and DE groups (n = 3 from three animals in each group). **B** The relative protein expression of PSD-95 and the corresponding protein band (n = 3). **C** Diagram of the proposed mechanism. *, **, and ***indicate *P* < 0.05, *P* < 0.01, and *P* < 0.001, respectively

The cAMP/PKA/CREB signaling pathway activates hippocampal synaptic plasticity, and is required for learning and memory function (Chen and Ganetzky 2012; Schreiber et al. 2020). The preservation of cognitive ability during aging depends on synaptic plasticity. As a result, we investigated whether exercise may promote synaptic plasticity and measured the levels of synaptic proteins in the hippocampus. By using immunofluorescence staining, we found that the average fluorescence intensity of SYP was higher in the DE group than in the DA group (Fig. 4A, *P* = 0.01). However, no difference in the average fluorescence intensity of SYP was observed in the DC group (Fig. 4A). We also analyzed the protein expression of PSD-95, which is associated with postsynaptic structure and function. The change in the expression of PSD-95 in the hippocampus was reversed in D-gal-induced aging model rats subjected to six weeks of exercise training (Fig. 4B, *P* = 0.04). Together, these findings indicate that exercise increased hippocampal synaptic plasticity, which had positive impacts on aging rats.

## Discussion

The degenerative changes caused by aging induces damage to hippocampal neurons, which affects learning and memory (Lombroso and Ogren 2008; Manabe et al. 2021). In this research, an aging model was well-established by continuous intraperitoneal injection of D-gal for six weeks. The rats in the DA group exhibited symptoms of aging and a significant decrease in body weight (Fig. S1). Cumulatively, our data show that exercise training exerts beneficial effects on a rat model of D-gal-induced aging by affecting hippocampal PDE-4 expression. We found that the mechanism underlying this beneficial effect of exercise may be PDE-4 DNA methylation. PDE-4 methylation can decrease the gene and protein expression of PDE-4, activate the cAMP/PKA/CREB pathway, improve hippocampal synaptic plasticity, and reverse cognitive impairments in aging animals. Our results identify a critical intracellular mechanism by which exercise training mediates cognitive function from an epigenetic perspective.

Our previous research established that downregulating the expression of PDE-4 can promote cognitive function (Shuling et al. 2017). To explore the potential mechanism, we assessed the relationship between PDE-4 and cognition from an epigenetic perspective. Several academic studies have shown that PDE-4 negatively regulates memory by impairing hippocampal neurogenesis (Bruel-Jungerman et al. 2005; Epp et al. 2007), while inhibition of PDE-4 can block cellular apoptosis in hippocampal neurons (Xiao et al. 2020). Previous studies have reported that PDE-4 knockdown alleviates memory deficits, rescues long-term potentiation (LTP) and ameliorates synaptic failure in AD (Shi et al. 2021).

As a specific hydrolase of cAMP, PDE-4 catalyzes the hydrolysis of the 3ʹ,5ʹ-phosphodiester bond of cyclic adenylate to generate inactive 5ʹ-nucleoside monophosphate. Recent studies revealed that PDE-4 inhibitor improves the learning and memory deficits via activating the cAMP/PKA/CREB signaling pathways (Peters et al. 2014; Shi et al. 2021). Consistent with (Kader et al. 2018), Our results demonstrate that exercise training causes PDE-4 hypermethylation at 5 sites in the hippocampus of D-gal-induced aging rats, resulting in PDE-4 gene silencing, and downregulation of PDE-4 mRNA and protein expression (Fig. 3A–C). The cAMP/PKA/CREB pathway was both positively and negatively regulated by the alterations in PDE-4 methylation and expression. In addition to increasing the expression of synapse-associated proteins and dendritic spine density, activated CREB also boosts the transcription and expression of related genes and promotes synaptic plasticity (Kandel 2012; Li et al. 2015). A previous study confirmed that exposure to young blood through heterochronic parabiosis ameliorates cognitive decline and increases the dendritic spine density and synaptic plasticity in the hippocampi of aging mice and that these changes are mediated by activation of CREB signal (Villeda et al. 2014). To validate these results, we performed immunofluorescence and immunoblotting to detect related synaptic structural proteins, including SYP, and PSD-95, which are important for synaptic plasticity. The results showed that exercise training increased the expression of associated synaptic structural proteins (Fig. 4A, B). Moreover, since a previous study has shown the role of PDE4 in hippocampal neurogenesis (Li et al. 2009), we believe that exercise-driven PDE4 methylation will improve learning functions via facilitating both neurogenesis and synaptogenesis. The current results support our findings suggesting a critical role for exercise training in promoting cognitive function by increasing PDE-4 methylation (Zhang et al. 2023). The current data further broaden our understandings for central effects of exercise, which leads to the neural recovery covering neuronal oxidative stress and synaptic plasticity. Those structural and functional enhancements help to explain the rescued spatial learning/memory impairments after the exercise training (Fig. 4C).

## Conclusions

In summary, our study demonstrates that the physical exercise promotes hypermethylation in the hippocampus and ameliorating synaptic dysfunction and cognitive impairment in a rat model of D-gal-induced aging. The effects of PDE-4 inhibitors, such as rolipram, were not investigated in this study. Thus, an additional work needs to be done to address this limitation in the future.

### Supplementary Information

Below is the link to the electronic supplementary material.

Supplementary file1 (DOCX 387 KB)

## Data Availability

The data presented in this study are available in article or supplementary material.
