# Supplementary material for: Effects of exercise-targeted hippocampal PDE-4 methylation on synaptic plasticity and spatial learning/memory impairments in D-galactose-induced aging rats
Source: Exp Brain Res. 2023 Dec 5;242(2):309–20. doi: 10.1007/s00221-023-06749-9 (PMC10805951; doi:10.1007/s00221-023-06749-9)

Supplementary materials

Fig. S1 Time course of the weekly changes in body weight in the different experimental groups over six weeks.


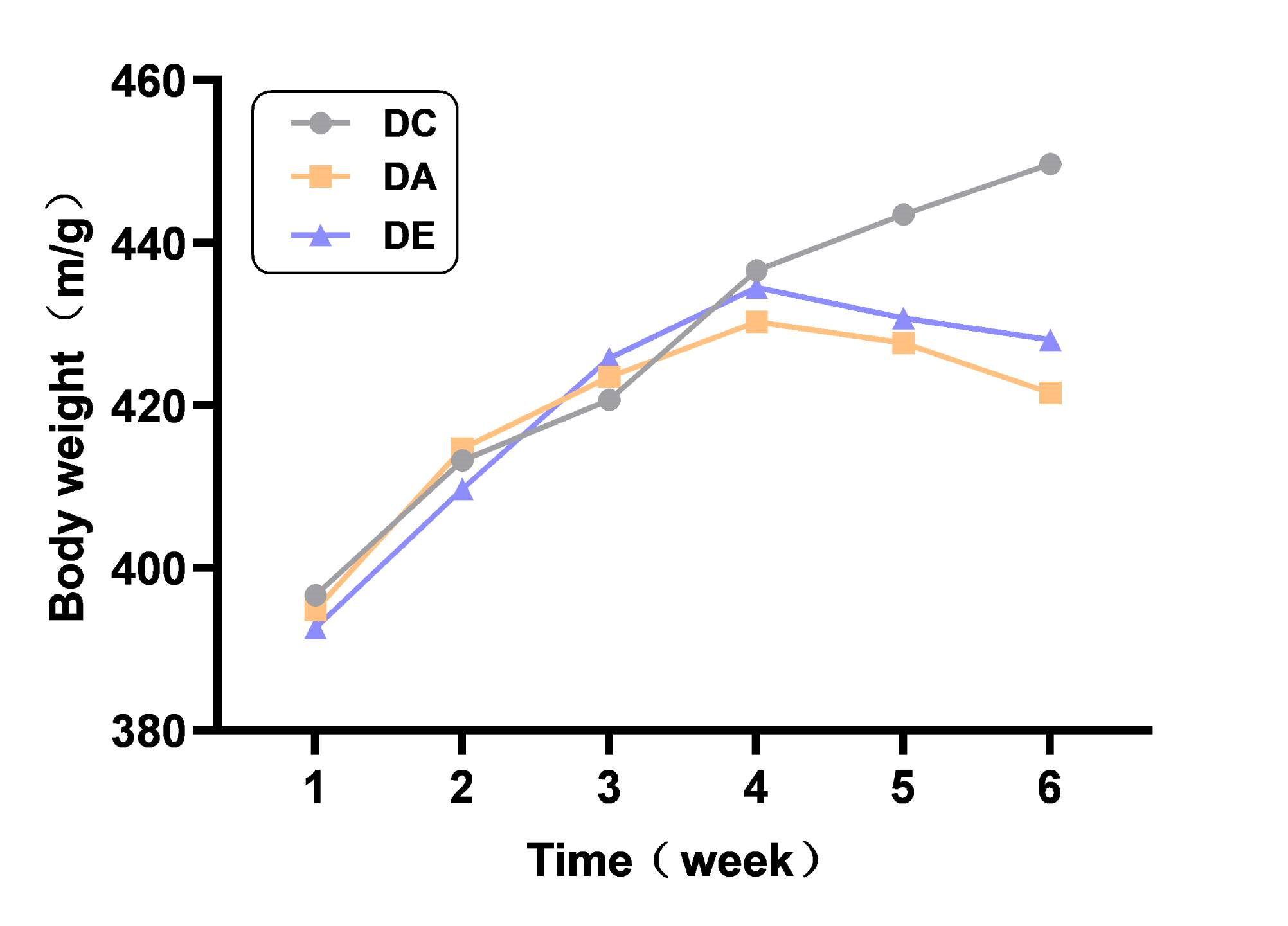

Supplement: Supplementary file 1 — Supplementary file1 (DOCX 387 KB) [file 221_2023_6749_MOESM1_ESM.docx]
